# Osteotomy combined with lateral ligament reconstruction in treating osteochondral lesion in patients with talar injury and varus ankle

**DOI:** 10.1097/MD.0000000000024330

**Published:** 2021-03-26

**Authors:** Mu Hu, Xiang-Yang Xu

**Affiliations:** Department of Orthopedics, Shanghai Ruijin Hospital North, Shanghai Jiaotong University School of Medicine, Shanghai, China.

**Keywords:** lateral ligament reconstruction, supramalleolar osteotomy, talar osteochondral lesion, varus ankle

## Abstract

This study aimed to investigate the therapeutic effects of osteotomy combined with lateral ligament reconstruction on the osteochondral lesion of patients with talar injuries and varus ankles.

Seventy five patients with talar injuries and varus ankles who received osteotomy combined with lateral ligament reconstruction for the osteochondral lesions from June 2008 to December 2014 were retrospectively reviewed. Patients were followed up for 32.4 ± 15.3 months after surgeries, and the AOFAS-AH score, VAS score and SF36 score were determined preoperatively and postoperatively. The iconographic data were compared preoperatively and postoperatively, including tibial anterior surface angle (TAS), TTS, TT, and tibial lateral surface angle (TLS) angles.

After surgeries, the AOFAS-AF score increased from 43.2 ± 8.1 to 82.1 ± 5.6, the VAS score decreased from 6.9 ± 2.3 to 1.8 ± 1.5, and the SF36 score increased from 48.7 ± 9.4 to 83.5 ± 6.2. TAS increased from 83.3 ± 5.1 to 90.3 ± 6.1, TTS increased from 70.3 ± 6.1 to 82.5 ± 5.4, TT decreased from 12.9 ± 6.1 to 6.9 ± 5.7, and TLS increased from 76.5 ± 4.1 to 81.2 ± 3.3 (*P* < .05).

Osteotomy combined with lateral ligament reconstruction is effective for the treatment of talar osteochondral lesion with varus ankle, which could relieve the arthritic symptoms induced by cartilage lesions. By correcting the force line on lower limbs and metapedes with osteotomy completely, the treatments on talar osteochondral lesion and lateral ligament reconstruction are the critical factors with better results.

## 1 Introduction

Traumatic arthritis, primary arthritis, inflammatory arthropathy, and infectious and neurotrophic arthrosis may cause ankle osteochondral lesions, and the traumatic arthritis has been the most common cause.^[1]^ Fifty percent of the patients with osteochondral lesions after ankle ligament injury will develop ankle varus deformity, while 25% will have ankle valgus deformity, accompanied with compensatory in subtalar joint. Valderrabano et al also found that 55% of patients with the osteochondral lesion had ankle varus deformity, while 8% developed ankle valgus deformity.^[2]^ The osteochondral lesion of these patients with end-stage ankle arthritis is serious, and may cause sustained clinical symptoms (such as spontaneous pain), functional limitation or even loss of the ankle joint, greatly influencing their quality of life.^[3]^ Glazebrook et al^[4]^ reported that the end-stage ankle osteochondral lesion would result in physiological and mental consequences, which were as serious as the end-stage hip osteoarthritis.

The varus deformity of the ankle may cause the abnormal stress distribution at the tibiotalar joint, cartilage metabolic disorder, and cartilage degeneration, damage, swelling, or exfoliation, which result in the osteoarthritis of the ankle joint.^[5]^ Therefore, the surgical treatment of the end-stage ankle arthritis with osteochondral lesion is important for the functional recovery of the ankle.

Recently, the ankle arthrodesis has been the most common treatment for the end-stage ankle arthritis.^[1,2]^ Although the arthrodesis is effective to relieve pain and recover the functions, the ankle joint movement becomes impossible, which may result in the arthritis adjacent to the joint with inflammations increasing.^[2,5,6]^ The artificial ankle has the advantages of preservation of joint movements and ankle function. More and more clinical evidence confirms the better therapeutic effects.^[7–9]^ However, the indications to treatment with prosthesis are complex.

The ankle osteotomy is one of the effective treatments that can maintain the joint function. By osteotomy on the distal tibia, intra-articular osteotomy, internally and externally double osteotomy and calcaneal osteotomy, the stress distribution of the ankle joint is rebalanced, the accumulated stress is relieved, the force line is recovered, and the progression of arthritis is delayed, which help to strive more time for stage II arthroplasty or arthrodesis as the final treatment of ankle arthritis. Middle to long term studies have shown the supramalleolar osteotomy may achieve favorable efficacy in the treatment of ankle arthritis.^[10]^

Herein, we retrospectively reviewed 75 patients with varus ankle arthritis who received ankle osteotomy combined with lateral ligament reconstruction. The clinical therapeutic efficacy of the ankle osteotomy combined with debridement and lateral ligament reconstruction was assessed; the causes of treatment failure and the complications were analyzed; the indications and standard procedures were summarized.

## 2 Materials and methods

### 2.1 Clinical characteristics

The clinical and imaging findings of 75 patients were retrospectively reviewed. There were 26 males and 49 females. Of them, 31 had left ankle injury and 44 had right ankle injury. The average age was 54.6 ± 7.4 years (Table 1). All the patients received ankle osteotomy combined with lateral ligament reconstruction for the varus ankle arthritis with osteochondral lesion in our hospital between June 2008 and December 2014. This study has been approved by the Ethics Committee of Shanghai Ruijin Hospital North, Shanghai Jiaotong University School of Medicine. The inclusion criteria were:

1. patients received ankle osteotomy combined with lateral ligament reconstruction;
2. patients with osteochondral lesion;
3. patients with varus ankle;
4. with clinical symptoms.

**Table 1:** Demographics of patients.

Characteristics	
Number of patients, n	75
Male: female, n (%)	26 (34.7%); 49 (65.3%)
Side (left; right), n (%)	31 (41.3%); 44 (58.7%)
Age at surgery, y	54.6 ± 7.4
Cause	
Ankle instability after lateral ligament injury	45 (60%)
Ankle fracture	18 (24%)
Neuromuscular varus deformity on the ankle	12 (16%)

The exclusion criteria were:

1. patients with end-stage ankle arthritis;
2. patients with other ankle surgeries;
3. regional infection;
4. patients had severe osteoporosis or large bone loss around ankle joint.

According to grading system reported by Takakura et al, the ankle arthritis was graded as follows: 0, parallel joint, without tilted tibiotalar or signs of arthritis;

1. parallel joint, without tilted tibiotalar, subchondral osteosclerosis or osteophyte;
2. tilted tibiotalar, varus or valgus, without subchondral contact;
3. tilted tibiotalar, varus or valgus, subchondral contact;
4. all joint relaxant accompanying with subchondral contact.^[11]^

All the patients had varus ankle deformity, grade 2 was found in 29, grade 3a in 25 cases, and grade 3b in 21 cases.

### 2.2 Preoperative preparations

**Figure 1:**
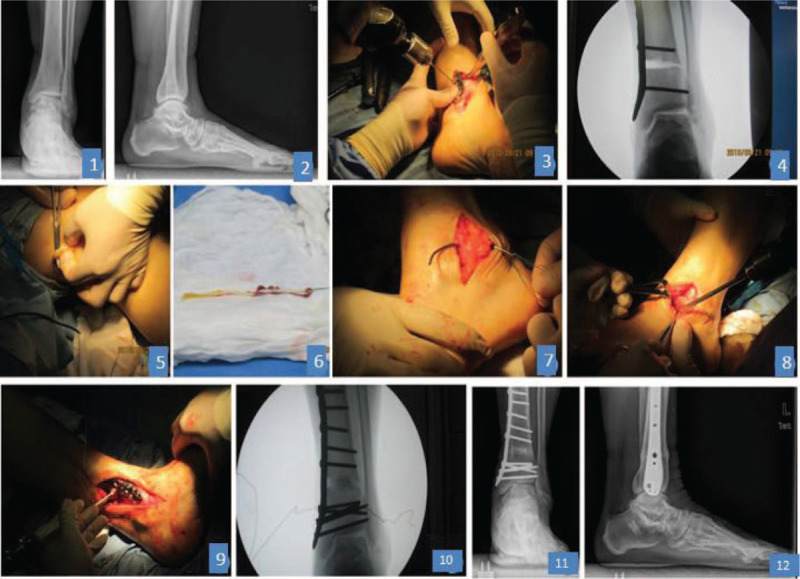
A 58-year female patient with left ankle injury 20 years ago; (A, B) grade 3a varus osteoarthritis before surgery; (C) supramalleolar osteotomy: internal open osteotomy on the distal tibia and fixation with the steel plate; (D) intraoperative situation; (E, F) collection of autogenous semitendinosus; (G bone tunnel construction on distal, tendon was leaded by an arc-needle to the tunnel; (H) the tendons were squeezed and the screws were fixed between the talar neck and the bone nodule to reconstruct the anterior tibiofibular and calcaneofibular ligaments; (I, J) the allogenic bone was embedded in the open osteotomy gap, and the fixed position of the allogenic bone was confirmed; (K, L) the medial ankle mortise was widened and the varus ankle arthritis was corrected.

The length of both legs was measured before osteotomy. The X-ray of the whole legs was done. If the patients had deformity on the force line of the proximal joints (such as knee). A line was drawn along the long axis and the facies articularis inferior to the tibias. Normally, the tibial anterior surface angle (TAS) is average 93°, and the tibial lateral surface angle (TLS) is 80°. According to the articular surface of the tibia, the sagittal point of rotation was confirmed as the axis of tibia (Fig. 1). The hindfoot view X-ray of Saltzman position was done and the force line was evaluated. The joints were carefully checked, and whether there were anchylosis, moving limitations and deformity and which could be recovered after surgery were further determined. According to the deformity, the degree of abnormal TAS and TLS, limb shortening and skin condition at the surgical site, the type of osteotomy (open or close) was determined. The calcaneal osteotomy was determined according to the force line and the deformity on X-ray.

### 2.3 Surgery

The patients firstly received debridement on the ankle and posterior soft tissues. The calcaneal tendon lengthening was performed according to the contracture, aiming to prepare for the talus reduction. The osteotomy was followed. Twenty six patients received supramalleolar and calcaneal osteotomy, 24 received supramalleolar osteotomy, 16 received supramalleolar and internal osteotomy, and 9 received calcaneal osteotomy. In addition, 60 patients received lateral ligament reconstruction according to the lateral ligament conditions after complete correction of force line, while 15 received external strengthening of the ligament. Thirty seven had cartilage grafting, 27 had microfracture, and 11 had cartilage debridement. The joint function before and after the surgery was evaluated with the AOFAS-HA score.

**Figure 2:**
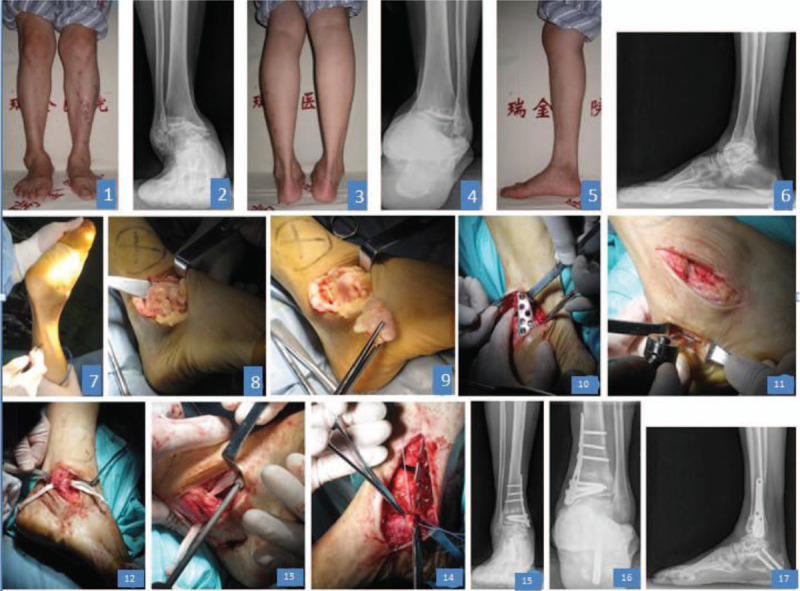
A 69-year female patient with right ankle injury for 18 years and aggravation for 5 years; (A–F) normal and lateral X-rays of right ankle and metaleg force line, grade 3b varus ankle arthritis, and metaleg varus deformity; (G) Achilles tendon was prolonged; (H–J) external osteophyte was cleaned, and the anterior talofibular ligament was loosened and dysfunctional; (J) open osteotomy of the internal distal tibia which was fixed by the steel plate; (K) calcaneus diagonal osteotomy was carried out and emigration, and 2 cannulated screws (6-7 mm in diameter) were fixed; (I) allogenic ligament was collected for reconstruction through the distal tibia bone tunnel; (M) lateral ligament and talus were fixed on the neck, and lateral calcaneus on body with compression screws (7 × 23 mm); (N) the internal lateral ligament was prolonged and sutured; (O–Q) normal and lateral X-rays indicating the correction of the varus ankle and metapodium.

The surgery was conducted as follows (Figs. 1 and 2): the patients lied in a supine position and received general or continuous epidural block anesthesia. The pneumatic tourniquets were used on the legs and the routine disinfection was carried out. An arc incision was made at the medial ankle to expose the medial ankle and distal tibia. The interior soft tissues were separated and the ligamentum deltoideum or spring ligament was prolonged if necessary. The proliferative synovium was removed and the osteophyte in talus, anterior and internal tibia was cut. Then, the external ankle was exposed. The synovium was cleaned and the osteophyte was cut. The achilles tendon were checked. If the axes tended to be internal, the calcaneal tendon was lengthened with three-point method. The open osteotomy was carried out at 3.5 cm away from the distal tibia, and the structural allografts were imbedded and fixed by the anatomic plates. X-ray was employed to confirm the reduction and the fixing placements, and the force line was checked. The external calcaneal osteotomy was carried out for the patients still with varus. The calcaneal diagonal osteotomy was carried out via a small incision and the achilles tendon reset conditions were checked. Two cannulated screws (6.5 mm in diameter) were used for fixation. The autogenous semitendinosus or allogeneic tendon was used to reconstruct the anterior tibiofibular and calcaneofibular ligaments. The grafting ligament between the talar neck and the bone nodule was fixed. The internal deltoid ligament was prolonged and reconstructed. The force line was checked again for the correction of the deformity and confirmation of the position after fixation. The wound was cleaned completely and closed. The ankle was immobilized at the neutral position with the U-shaped plaster.

### 2.4 Clinical analysis

The AOFAS-AH, VAS, and SF36 scores were determined before and after operation to evaluate the objective and subjective appearances.

### 2.5 Imaging examination

X-ray of the loading area of the ankle at standing position, including the anterior and posterior positions, ankle mortise, lateral position and Saltzman position. The TAS, TTS, TT (TT = TAS-TTS), TLS, and MoA at Saltzman position were measured before and after operation and compared.

### 2.6 Statistical analysis

Statistical analysis was performed with SPSS version 17.0. Wilcoxon signed-rank was used to compare the scores of AOFAS-AG, VAS and SF36, TAS, TTS, TT, TLS, and MoA before and after operation. A value of *P* < .05 was considered statistically significant.

## 3 Results

The average follow-up time was 32.4 ± 15.3 months. After surgery, the AOFAS-AF score increased from 43.2 ± 8.1 to 82.1 ± 5.6, the VAS score decreased from 6.9 ± 2.3 to 1.8 ± 1.5, and the SF36 score increased from 48.7 ± 9.4 to 83.5 ± 6.2 (*P* < .05) (Table 2).

**Table 2:** AOFAS-AG, VAS and SF-36 scores before and after surgery.

	AOFAS-AH	VAS	SF-36
Preoperative	43.2 ± 8.1	6.9 ± 2.3	48.7 ± 9.4
Postoperative	82.1 ± 5.6	1.8 ± 1.5	83.5 ± 6.2
*P*	<.05	<.05	<.05

There were 45 patients with varus ankle arthritis after lateral ligament injury, 18 with ankle varus deformity after ankle fracture, and 12 with neuromuscular varus deformity on the ankle. The average time from the ankle injury to the surgery was 12.7 ± 10.2 years, and the average duration of following-up was 32.4 ± 15.3 months.

After surgery, the TAS increased from 83.3 ± 5.1 to 90.3 ± 6.1, the TTS increased from 70.3 ± 6.1 to 82.5 ± 5.4, the TT decreased from 12.9 ± 6.1 to 6.9 ± 5.7, and the TLS increased from 76.5 ± 4.1 to 81.2 ± 3.3 (*P* < .05) (Table 3).

**Table 3:** TAS, TTS, TT and TLS before and after surgery.

	TAS	TTS	TT	TLS
Preoperative	83.3 ± 5.1	70.3 ± 6.1	12.9 ± 6.1	76.5 ± 4.1
Postoperative	90.3 ± 6.1	82.5 ± 5.4	6.9 ± 5.7	81.2 ± 3.3
*P*	<.001	<.001	<.001	.127

## 4 Discussion

In our study, more patients (45/75) had lateral ligament injury and consequent ankle arthritis. The long term arthritis and the unstable ankle may cause talus and calcaneus varus, which lead to the regional asymmetrical ankle arthritis of grade 2-3b, requiring osteotomy. The patients with stage 4 ankle arthritis, who developed end-stage ankle arthritis due to ankle fracture were suitable for ankle fusion or total ankle replacement (TAR).

The ankle fusion was considered as the standard surgery for the serious and end-stage ankle arthritis. However, the fusion will make the ankle movement impossible, and the ankle arthritis in the adjacent joints will result in pain, deformity and dysfunction.^[12–14]^ It has been reported that the incidence of ankle arthritis is 70% after ankle fusion in 10 years.^[12–14]^ TAR is one of the treatments for the end-stage ankle arthritis, but it is not suitable for the young or heavy patients with active joint movement or complications. The grade 1 to 3 ankle arthritis, especially in those with chronicity and instability due to lateral ankle injury, usually has concomitant osteochondral lesion on the internal ankle. For the regional ankle arthritis, the ankle fusion or TAR is over treated, which may cause the loss of complete cartilage. Therefore, the ankle osteotomy is suitable for the regional ankle arthritis, which has been widely used in clinical practice.^[10,15–17]^ Supramalleolar osteotomy is usually employed to treat the early to mid-stage ankle arthritis.^[18]^

Lee et al reported that, when the incline angle of the talus was smaller than 7.3°, the supramalleolar osteotomy was the preferred treatment.^[15]^ Tanakal et al proposed that the incline angle of the talus could be broadened to 10°.^[11]^ In our study, the incline angle of the talus was average 14.1 ± 6.2°, which suggested a more serious condition as compared to previously reported. For these patients, the supramalleolar osteotomy may be a preferred treatment with favorable efficacy.

A majority of patients in China have the requirement of ankle preservation in order to improve the life quality. Additionally, the physicians with abundant training and clinical experiences were less in China. The lacking of advanced ankle prosthesis, the less use of ankle prosthesis in China is a big problem. Therefore, for the patients with talar incline angle lager than 10°, the clinicians should objectively assess the disease condition to preserve the ankle, in order to maintain the range of motion larger than 30°, as well as the consideration of the patients.

In our study, most of patients had chronic and instable lateral ligament injury, which led to varus ankle arthritis, and the long-lasting arthritis caused the supramalleolar and subcondylar deformity. Thus, the supramalleolar and subcondylar ankle osteotomy was needed to correct the varus and deformity completely. The external lateral ligament was with thinning, loosing and dysfunction for long time of internal varus. The internal lateral and capsular ligament were with contracture, which would become loose or lose the function for fracture after cleaning osteophyte. Therefore, the lateral ligament reconstruction was needed to maintain the neutral position of the ankle for the recovery of the ankle stability.

For the patients with unstable lateral injury and with more tissues, the Brostrom-Gould ligament repairing and strengthening were suitable. For the patients with less lateral ligament tissues, which could not assure the ankle movement, or the patients requiring large range of motion or with higher BMI, the lateral ligament reconstruction is usually needed. The semitendinosus tendon or allogenic ligament was chosen. The compression screws were used to fix the talar neck and calcaneal tuberosity. The anterior talofibular and calcaneofibular ligaments were reconstructed. For the varus ankle arthritis as a sequela of bone fracture and the shorter process, the deformity and pain still sustained, but the patients were diagnosed early and received the single supramalleolar osteotomy to correct the deformity. During the surgery, the external lateral ligament was found to be complete, and the lateral ligament was less influenced. After the surgery, the talar tilt was 6.9 ± 5.7°, which was still larger than 5°. For most of the patients with lateral ligament injury combined with chronicity and instability, which resulted in varus ankle arthritis, long lasting arthritis and serious deformity and varus, external lateral ligament reconstruction was needed to maintain the neutral position of the ankle to prevent the recurrence of deformity. After the position was altered, the ankle concentrated the stress, the inflammatory tissue and osteophyte were cleaning and the ankle stability was reconstructed, the symptoms were improved after the correction of the deformity though the talar tilt was still larger than 5° on the imaging.

In conclusion, ankle osteotomy combined with lateral ligament reconstruction is effective to treat the talar osteochondral lesion with varus ankle, and can relieve the arthritic symptoms. By correcting the force line on the lower limbs and metapedes with osteotomy completely, the treatment for talar osteochondral lesion and the ligament reconstruction may achieve better outcomes.

## Author contributions

**Conceptualization:** Mu Hu, Xiang-Yang Xu.

**Data curation:** Mu Hu.

**Formal analysis:** Mu Hu.

**Investigation:** Mu Hu.

**Methodology:** Mu Hu.

**Resources:** Mu Hu, Xiang-Yang Xu.

**Software:** Mu Hu.

**Visualization:** Xiang-Yang Xu.

**Writing – original draft:** Mu Hu.

**Writing – review & editing:** Xiang-Yang Xu.
